# Flow Behavior through Porous Media and Displacement Performance of a SILICA/PAM Nanohybrid: Experimental and Numerical Simulation Study

**DOI:** 10.1021/acsomega.3c07476

**Published:** 2024-02-07

**Authors:** Laura M. Corredor, Carlos Espinosa, Claudia L. Delgadillo, Sebastian Llanos, Rubén H. Castro, Henderson I. Quintero, Maria Carolina Ruiz Cañas, Arnold Rafael Romero Bohorquez, Eduardo Manrique

**Affiliations:** † Instituto Colombiano del Petróleo, ECOPETROL S.A., Piedecuesta 681011, Colombia; ‡ Cooperativa de Tecnólogos e Ingenieros de la Industria del Petróleo y Afines, Girón 681012, Colombia; § Grupo de Investigación en Química Estructural, Departamento de Química, Universidad Industrial de Santander, Bucaramanga 680006, Colombia; ∥ Meridian Consulting, Bogotá 110231, Colombia

## Abstract

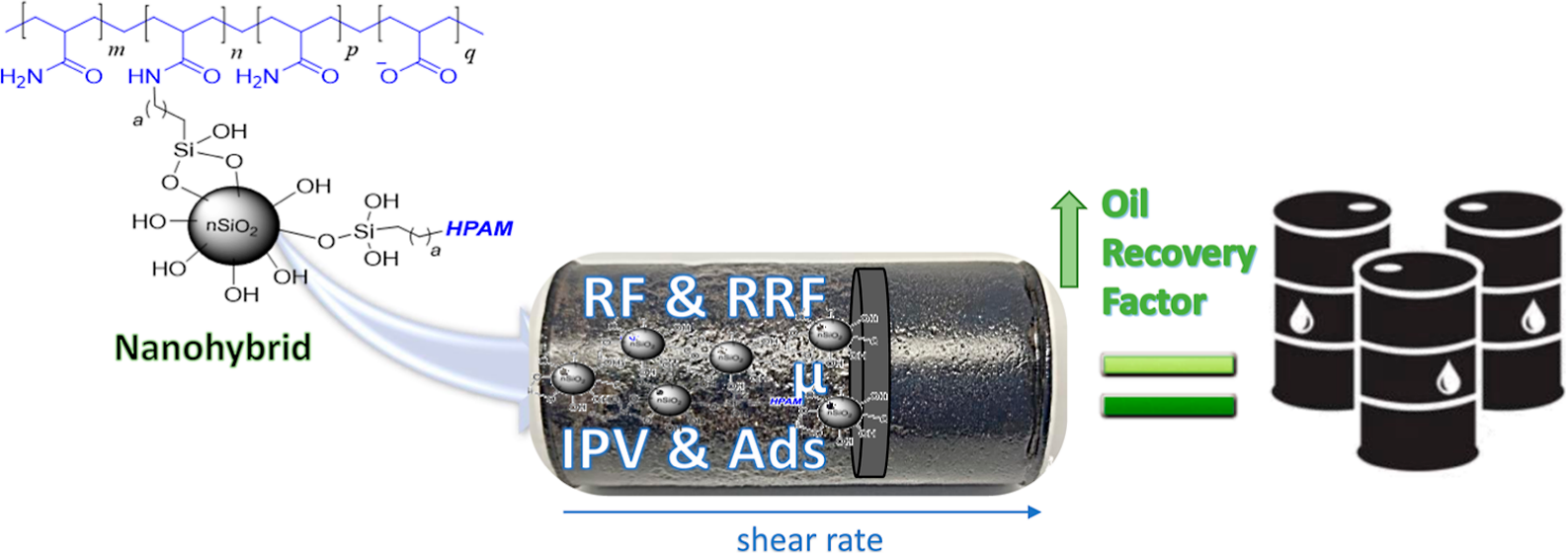

Nanoparticles (NPs) have been proposed as additives to improve the rheological properties of polymer solutions and reduce mechanical degradation. This study presents the results of the retention experiment and the numerical simulation of the displacement efficiency of a SiO_2_/hydrolyzed polyacrylamide (HPAM) nanohybrid (CSNH-AC). The CSNH-AC was obtained from SiO_2_ NPs (synthesized by the Stöber method) chemically modified with HPAM chains. Attenuated total reflection–Fourier transform infrared spectroscopy, field emission gun–scanning electron microscopy, X-ray diffraction, and thermogravimetric analysis were used to characterize the nanohybrid. The injectivity and dynamic retention tests were performed at 56 °C in a sandstone core with a porosity of ∼26% and a permeability of 117 and 287 mD. A history matching of the dynamic retention test was performed to determine the maximum and residual adsorption, IPV, and residual resistance factor (RRF). A laboratory-scale model was used to evaluate the displacement efficiency of CSNH-AC and HPAM through numerical simulation. According to the results, the nanohybrid exhibits better rheological behavior than the HPAM solution at a lower concentration. The nanopolymer sol adsorption and IPV (29,7 μg/g_rock_, 14,5) are greater than those of the HPAM solution (9,2 μg/g_rock_, 10), which was attributed to the difference between the rock permeabilities used in the laboratory tests (HPAM: 287 mD and CSNH-AC: 117 mD). The RF of both samples gradually increases with the increase in shear rate, while the RRF slightly decreases and tends to balance. However, the nanopolymer sol exhibits greater RF and RRF values than that of the polymer solution due to the strong flow resistance of the nanohybrid (higher retention in the porous media). According to the field-scale simulation, the incremental oil production could be 295,505 and 174,465 barrels for the nanopolymer sol and the HPAM solution, respectively (compared to waterflooding). This will represent an incremental recovery factor of 11.3% for the nanopolymer sol and 6.7% for the HPAM solution.

## 1 Introduction

Several important characteristics must be evaluated before injecting a polymer into a reservoir formation, which include low cost, suitable viscosity, salinity and temperature tolerance, mechanical stability, low retention, and good injectivity.^1−4^ Polymer injectivity determines how easily a polymer solution can be injected and propagated through a reservoir formation.^5^ It is a critical characteristic because a reduction in injectivity can affect the cash flow of a polymer flooding project due to high pumping costs or delays in oil production.^6^ Polymer rheology and retention are the main factors that reduce injectivity. Polyacrylamide solutions have exhibited a dilatant behavior when propagating in porous media due to their elastic character,^7−10^ which increases resistance to flow.^11^ However, if the stretch rates that cause the dilatant behavior are high enough, then polymer chains can suffer mechanical degradation, yielding viscosity loss.

Polymer retention includes mechanical entrapment and adsorption.^12−17^ Mechanical entrapment occurs when polymer molecules are large relative to the size of pores.^2,18,19^ Some mechanisms observed in mechanical entrapment are hydrodynamic retention,^15,20,21^ bridging adsorption,^22,23^ and trapping on dead-end pores.^24^ Hydrodynamic retention is caused by osmotic forces, which temporally trap polymer molecules in stagnant regions of porous media.^25,26^ Adsorption occurs due to interaction between polymer molecules and the rock surface (especially between the polar groups of polymers and polar points available on the rock surface).^27,28^ Adsorption affects the solution concentration and effectiveness of the mobility control at the displacement front because the polymer is removed from the injected fluid. The amount of polymer adsorbed strongly depends on polymer concentration,^29,30^ polymer charge,^31^ permeability,^7,17,32^ clay and iron content,^33,34^ salinity, and pH.^35,36^

Static and dynamic methods are used to measure polymer adsorption in laboratory-scale experiments. In the static method, the polymer concentration is measured before and after soaking sand or crushed rock samples in the polymer solution. Polymer adsorption is determined by dividing the loss of mass from the solution by the weight of the sand or crushed rock sample. This method is simple and inexpensive. However, the results may not represent the field values because the surface area and the minerals exposed to the polymer may be different from those available in dynamic experiments,^29^ the wettability of the crushed rock may be different from that of the reservoir rock,^37^ and the polymer that can be mechanically entrapped is not measured.^33^

There are different methods for measuring the adsorption under dynamic flow conditions. In the first method, a polymer solution is injected at a constant frontal advance velocity into a linear core or sand pack until the normalized effluent concentration reaches unity. In the second method, polymer injection is switched to water or brine injection after the normalized effluent concentration reaches unity and the mobile polymer is displaced from the pore space.^29^ Polymer retention in both methods is determined by the material balance. Another method is the one proposed by Loetsch et al.,^38^ Hughes et al.,^39^ and Osterloh and Law.^40^ In this method, a slug of polymer solution is injected into a linear core or sand pack with a tracer. After the normalized concentration for both polymer and tracer reaches unity, the injection is switched to brine or water. Subsequently, the second slug of the polymer is injected with a tracer. Polymer retention and inaccessible pore volume (IPV) are determined by using the front part of the effluent curves during the two injection stages. IPV is calculated as the difference in area between the polymer-breakout curve and the tracer-breakout curve during the second injection stage. In the last method (concentration profile method), two polymer slugs are injected following the same procedure as explained previously. Adsorption is calculated from the cutoff values of the normalized concentration at 0.5 for both polymer slugs. IPV is calculated by 1 minus the value of the normalized concentration at 0.5 of the second polymer slug.^26^

Experimental studies have shown that incorporating nanoparticles (NPs) can improve the rheological properties of the displacing fluid^41−46^ and reduce the degradation effect of salinity and temperature on polymer solutions^45,47−53^ and the polymer adsorption onto the rock surface.^54−56^ According to results presented by Abdullahi et al.^47^ and Maghzi et al.,^48^ the NPs prevent the electrical shielding effect caused by the presence of cations in the polymer solution because the ion–dipole interactions occur between the cations and the oxygen on the NP surface instead of the cations and the amide groups of the polymer molecules. The improvement of the rheological properties of hydrolyzed polyacrylamide (HPAM) solutions following NP incorporation has been attributed to the formation of a three-dimensional network in which different polymer chains are cross-linked by NPs.^57−59^ Few studies on the flow behavior of polymer nanohybrids through porous media have been reported. For this reason, more investigations are needed to improve our knowledge of the underlying enhanced oil recovery (EOR) mechanisms of polymer nanohybrids.

For the reasons stated above, the aim of this study is to evaluate the effect of surface-modified SiO_2_ NPs on the flow behavior in porous media and the oil displacement efficiency of the HPAM solution. Displacement tests were performed to quantify the polymer retention, the IPV, and the resistance and residual resistance factors (RRFs) of the HPAM solution and the nanopolymer sol. The history matching of the dynamic retention test was performed by using the STARS module of CMG. The history matching parameters were used to predict the displacement efficiency for injecting 0.3 PV of 550 ppm of nanopolymer sol and 750 ppm of HPAM solution.

## 2 Methodology

### 2.1 Materials

The chemicals used for the preparation of the SiO_2_ NPs were tetraethoxysilane (TEOS, 98%, Sigma-Aldrich, USA), ethanol (EtOH, 96%, Merck Millipore, USA), and ammonium hydroxide (28–30 wt % solutions of NH_3_ in water, purity, Merck Millipore, USA). 3-Aminopropyltriethoxysilane (APTES, 99%, Sigma-Aldrich, USA) and HPAM (25–35% hydrolysis degree, MW ≈ 20 × 10^6^ daltons) were used to modify the SiO_2_ NPs. The solvents used in this reaction were tetrahydrofuran (THF, 99.9%, Sigma-Aldrich, USA), 2-propanol (C_3_H_8_O, >99.8%, Merck Millipore, USA), and sulfuric acid (H_2_SO_4_, 97%, Merck Millipore, USA).

The injection and formation brine were prepared with sodium chloride (NaCl, 99.5% pure, Merck Millipore, USA), potassium chloride (KCl, 99.5% pure, Merck Millipore, USA), magnesium chloride (MgCl_2_·6H_2_O, 99% pure, Merck Millipore, USA), and calcium chloride (CaCl_2_·2H_2_O, 99% pure, Merck Millipore, USA). The sandstone cores, with a length of ∼6 cm and a diameter of 3.81 cm, were supplied by Ecopetrol S.A. The tracer used for the retention tests was potassium thiocyanate (KSCN, 99.7% pure, J.T Baker).

### 2.2 Nanohybrid Synthesis

The detailed synthesis of the nanohybrid used in this work has been reported previously by the authors.^60^ The SiO_2_ NPs were prepared by adding tetraethyl orthosilicate (TEOS, 1 mL) under vigorous stirring to a solution of ammonium hydroxide and ethanol (1:5 ratio) at 90 °C. After 3 h, the SiO_2_ NPs were recovered by centrifugation and dried for 24 h at 90 °C. The SiO_2_ NPs were modified with APTES (nSiO_2_-APTES) following the procedure proposed by Chen et al. (2009).^61^ The nanohybrid (CSNH-AC) was obtained by dispersing 2 g of nSiO_2_-APTES in a THF/water solution at 400 rpm and then adding 3 g of HPAM powder. The reaction was carried out at room temperature for 24 h. Thereafter, CSNH-AC was recovered by centrifugation and washed with 2-propanol. Finally, the product was dried at 60 °C for 24 h.

### 2.3 Nanohybrid Characterization

The size and morphology of the CSNH-AC were characterized through field emission gun–scanning electron microscopy (FEG–SEM) (QUANTA FEG 650 model, Thermo-Fisher Scientific, USA) at a high vacuum and an accelerating voltage of 25 kV. The X-ray diffraction pattern (XRD), used for structural analysis, was performed with a Bruker D-8 A25 DaVinci X-ray diffractometer (D8 ADVANCE, Bruker, Billerica, MA, USA) with CuKα radiation and a LynxEye detector at a voltage of 40 kV. FTIR spectra were collected on a Bruker Tensor 27 FTIR spectrometer (Alpha, Bruker, USA). Thermogravimetric analysis (TGA) was performed using a TA2050 TGA analyzer (TA Instruments, INC., USA). For the TGA measurements, a mass of 5 mg of the nanohybrid or the HPAM was heated from 25 to 800 °C at a heating ramp of 10 °C/min in a nitrogen atmosphere.

### 2.4 Fluid Preparation and Filtration

The formation and injection brine composition are presented in Table 1. Each brine was filtered through a 5.0 μm MCE membrane filter (Merck Millipore, USA) before use. The formation brine was employed in the core saturation and permeability measurements, while the injection water was used for the preparation of the nanopolymer sols and the polymer solutions. For this, a mass of 5 g of HPAM powder or nanohybrid was added into the injection water to prepare the stock solutions of 5000 ppm, respectively. Each sample was stirred at 200 rpm for 48 h before dilution into the required concentration. 30 ppm of KSCN were dissolved into the polymer solution or the nanopolymer sol to determine IPV and adsorption.

**Table 1:** Formation and Injection Brine Composition

salt	formation brine composition, g/L	injection brine composition, g/L
NaCl	8.648	2.348
KCl	0.037	0.011
MgCl_2_·6H_2_O	0.834	0.212
CaCl_2_·2H_2_O	1.673	0.432

### 2.5 Rheology of the Nanopolymer Sol and Polymer Solution

The flow curves of the nanopolymer sols and the polymer solutions were measured on an MCR502 rheometer (Anton Paar, Austria) with concentric cylinder geometry (measuring bob and measuring cup had radii of 13.329 and 14.463 mm, respectively) over the range 4–424 s^–1^. A strain amplitude of 1% was selected to ensure the samples fell within the linear viscoelastic region. The rheological behavior of the samples was well described by the Carreau–Yasuda model.^29^ The uncertainty of the reported value remained between ±1 and 4%.

### 2.6 Core Flooding Tests at 100% Sw

The properties of the sandstone plugs are listed in Table 2. These properties were measured by following the procedures described by McPhee et al.^62^ All tests were performed at 56 °C because it is the reservoir temperature of the Colombian field selected to evaluate the performance of the synthesized nanohybrid. The polymer solutions and nanopolymer sols were filtered and preheated before injection. For the preshearing process, 300 mL of sample were pressurized with nitrogen and passed through a capillary (ID 1/8″).

**Table 2:** Properties of the Sandstone Plugs Used for the Retention Tests

properties	plug 1	plug 2
depth, ft	4450.25	4456.33
length, cm	6.11	6.15
diameter, cm	3.81	3.81
pore volume, cm^3^	17.5	17.9
grain volume, cm^3^	49.1	49.5
porosity, %	26.1	26.4
Klinkenberg permeability, mD	117	287
rock type	2	2
sample	CSNH-AC	HPAM

#### 2.6.12.7.1 Resistance Factor and Residual Resistance Factor

First, the sandstone core plugs were vacuumed and saturated with formation brine. After that, the plug was mounted in the setup, the formation brine was injected at different flow rates (0.067, 0.167, 0.333, and 0.5 mL/min), and corresponding pressure drops were recorded. The absolute permeability was calculated by Darcy’s law. The salinity of the plugs was changed by injecting different formation/injection ratios until the plugs were fully saturated with the injection brine. Then, the brine injection continued at 0.067, 0.167, 0.333, and 0.5 mL/min, and the corresponding pressure drops were recorded. Second, the polymer solutions (750 and 950 ppm) or the nanopolymer sols (550 and 750 ppm) were injected at the same flow rates used in the previous step, followed by brine injection. All the stable pressure drops were recorded and used to calculate the RF and the RRF, which are defined as^13^
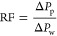
1
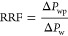
2where Δ*P*_w_ is the pressure drop during brine injection, Δ*P*_wp_ is the pressure drop during brine injection after polymer flooding, and Δ*P*_p_ is the pressure drop during polymer or nanopolymer sol injection.

#### 2.6.2 Dynamic Polymer Adsorption and IPV

The material balance method was used to measure the adsorption and IPV of the 750 ppm polymer solution and the 550 ppm nanopolymer sol. For this, each sample with 30 ppm of KSCN tracer was injected (until *C*/*C*_o_ on the effluents was equal to 1), followed by injection of brine (until polymer concentration on the effluents was close to zero). All fluids were injected at a rate of 0.067 mL/min. The effluents were collected to determine the KSCN, HPAM, and CSNH-AC concentrations through UV–vis analysis (DR5000, Hach, USA). For the UV–vis measurements, two 1 mL aliquots of the effluents were taken and treated with iron chloride hexahydrate (FeCl_3_·6H_2_O, Merck, USA) to determine the KSCN concentration and with sodium hypochlorite and glacial acetic acid to determine the HPAM and CSNH-AC concentrations.^63^ The procedure was repeated for the second batch of the polymer/nanopolymer sol and tracer solution. IPV and adsorption were calculated from eqs 3 and 4.^26^ The shear rate (γ̇) in porous media was calculated from eqs 5 and 6.^64,65^ Finally, the effective viscosity of the polymer solution in porous media was determined from eq 7.^66^
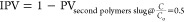
3
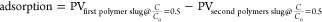
4
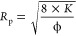
5
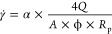
6
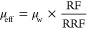
7where *C* is the polymer concentration in the effluent, *C*_o_ is the initial polymer concentration, *Q* is the flow rate (cm^3^/min), *A* is the surface flow area of the porous media (cm^2^), ϕ is porosity (fraction), *K* is the absolute permeability (cm^2^), *R*_p_ is the porous radius (cm), α is the formation shape factor which is assumed 1 (dimensionless) for the sandstone plugs, μ_eff_ is the effective viscosity of polymer (cP), and μ_w_ is the viscosity of water (cP).

The concentrations of the nanopolymer sol and the HPAM solution were selected to reach mobility ratios close to one (1.2 and 1.6, respectively) to minimize the viscous fingering in the core flooding tests. The mobility ratios were calculated from eq 8
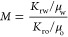
8where *K*_rw_ is the water-effective permeability, *K*_ro_ is the oil-effective permeability, μ_w_ is the water viscosity, and μ_o_ is the oil viscosity.

### 2.7 Numerical Simulation

**Figure 1:**
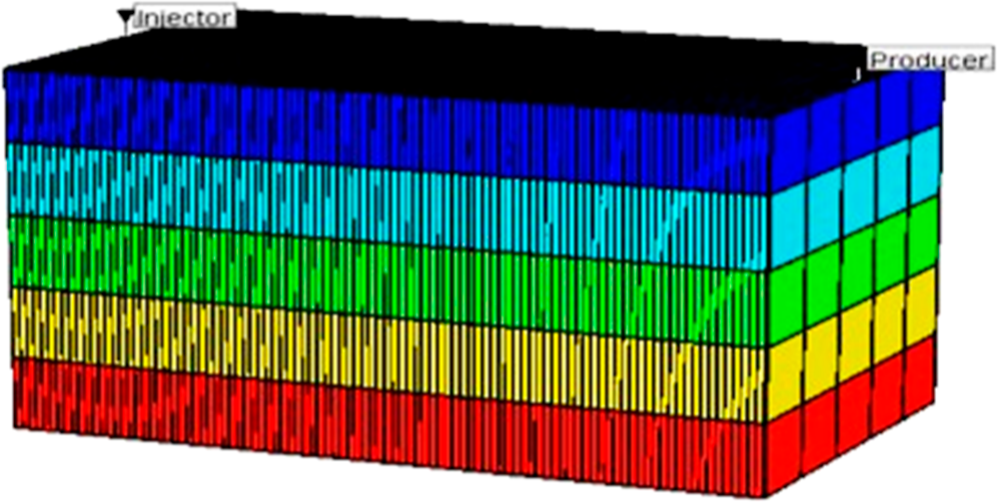
Model used to simulate the laboratory experiments.

The numerical simulation was performed using a laboratory-scale model built-in commercial software (CMG STARS). Also, the CMOST module was used to perform the history matching of the laboratory tests, combining advanced statistical analysis and machine learning. The fundamental grid dimensions were 100 × 5 × 5 for *X*, *Y*, and *Z*, respectively, and the total number of blocks was 2500. The properties of each model are summarized in Table 3. Some of these data correspond to the results obtained from the rheological and rock-fluid experiments for the nanohybrid sol and the polymer solution on the laboratory scale. The producer and injector wells were placed at the edge of the numerical grid, representing the inlet and outlet of the core holder. Although laboratory cores physically have cylindrical dimensions, the numerical models were built in Cartesian coordinates by adjusting the surface flow area and the pore volume (Figure 1).

**Table 3:** Parameters Used for the History Matching of the Numerical Simulation Model

properties	parameter	model 1	model 2	unit
core description	length	6.11	6.15	cm
	area open to flow^a^	11.4	11.4	cm^2^
	pore volume	17.5	17.9	cm^3^
	porosity	26.1	26.4	%
	absolute permeability^b^	117	287	mD
fluid properties	oil viscosity	43	cP	
	connate water viscosity	0.5	cP	
	CSNH-AC/HPAM viscosity—7.3 s^–1^	10.9	8	cP
rock-fluid properties	*K*_ro_ (Swi)	0.75	0.75	
	*K* (Sor)	1	1	
	residual oil saturation	35	35	%
	polymer adsorption	29.7	9.2	μg/g
	RRF	12	1.3	
	IPV	14.5	10	%
initial conditions	pressure	200	psi	
	temperature	56	°C	
	oil saturation	0	0	%
	water saturation	100	100	%
boundary conditions	injected fluid	CSNH-AC	HPAM	
	injection rate	0.067, 0.167, 0.333, 0.5	mL/min	
	CSNH-AC/HPAM concentration	550	750	ppm
	KSCN concentration	30	30	ppm

a Circular core section approximated to a square one with the same area open to flow.

b Considered to be the effective permeability to water.

The history matching provides a considerable understanding of the transport mechanisms. For this reason, the model input data was adjusted until the minimum difference between the results of the simulated model and the laboratory data was obtained. The methodology is based on the correct representation of the phenomena occurring during core flooding tests and the adjustment of some uncertain properties.^67^ It is a typical inverse problem where the result is known (laboratory production and pressure history), and the input parameters that allow the model to obtain this result must be determined. In this case, the input parameters adjusted were the permeability reduction factor, dynamic adsorption, and IPV. These parameters were selected as a result of the sensitivity analysis because they had the greatest impact on the objective functions of the history-matching process.

Once the best match was obtained, the new values of the fitting parameters were used to predict the displacement efficiency for the CSNH-AC and HPAM solutions in the same laboratory-scale model to evaluate the performance of both products under the same conditions. Oil recovery by waterflooding was used to represent a typical base scenario on the laboratory scale, followed by chemical flooding.

**Figure 2:**
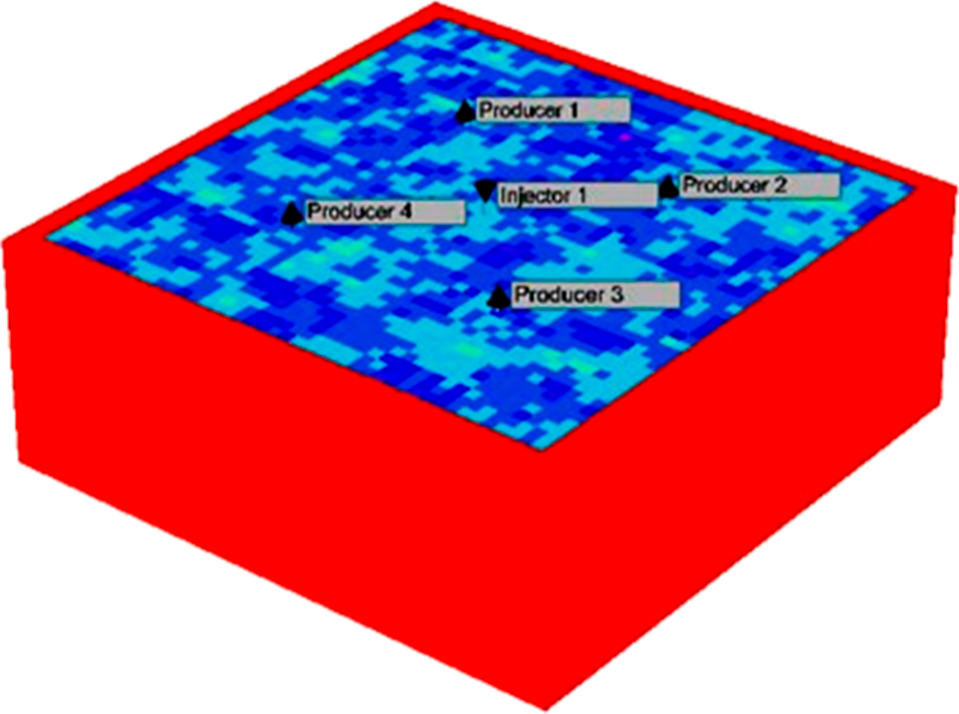
Sector model used for the numerical simulation of the nanopolymer sol and HPAM solution injection.

To evaluate the volumetric sweep efficiency of the CSNH-AC and HPAM injection on a field scale, an inverted 5-point injection pattern was built in a box model (Figure 2). The fundamental grid dimensions were 47 × 47 × 10 for *X*, *Y*, and *Z*, respectively, and the total number of blocks was 22,090. The PVT properties of water and dead oil are listed in Table 4. The porosity and permeability were defined through geostatistics. The pattern has an area of 20 acres, a pore volume of 3.75 million barrels, and a volume of oil in place of 2.62 million barrels. One injection well at the center of the pattern area was controlled by 600 BPD as the maximum injection rate and 3000 psi as the maximum bottom hole pressure. Four producer wells around the injector were regulated by 1000 BPD as the maximum liquid rate and 1000 psi as the minimum bottom hole pressure. The chemical injection was evaluated as a tertiary recovery method by injecting a 0.1 PV slug followed by chase water. The goal of injecting a small slug was to demonstrate that the performance of the CSNH-AC is better than that of the HPAM under this condition.

**Table 4:** Model Properties Used for Numerical Forecasting

properties	parameter	model	unit
model description	area	20	acre
	gross thickness	300	ft
	pore volume	3.75	MMSTB
	OOIP	2.62	MMSTB
fluid properties	oil viscosity	16	cP
	API gravity	27.7	
	formation volume factor	1.13	RB/STB
	gas specific gravity	0.633	
	solution gas-oil ratio	226	SCF/STB
	connate water viscosity	0.5	cP
rock-fluid properties	*K*_ro_ (Swi)	1	
	*K*_rw_ (Sor)	0.3	
	residual oil saturation	35	%
	initial water saturation	30	%
	HPAM residual adsorption	98	μg/g
	RRF for HPAM	1.25	
	IPV for HPAM	7.78	%
	CSNH-AC residual adsorption	17	μg/g
	RRF for CSNH-AC	10.4	
	IPV for CSNH-AC	7.37	%
initial conditions	pressure	1950	psi
	temperature	125	°F
	oil saturation	0	%
	water saturation	100	%
boundary conditions	injected Fluid	0.1	PV
	injection rate	600	BPD
	CSNH-AC/HPAM concentration	550/850	ppm
	KSCN concentration	30	ppm

## 3 Results and Discussion

### 3.1 Nanohybrid Characterization

#### 3.1.1 SEM and XRD Results

**Figure 3:**
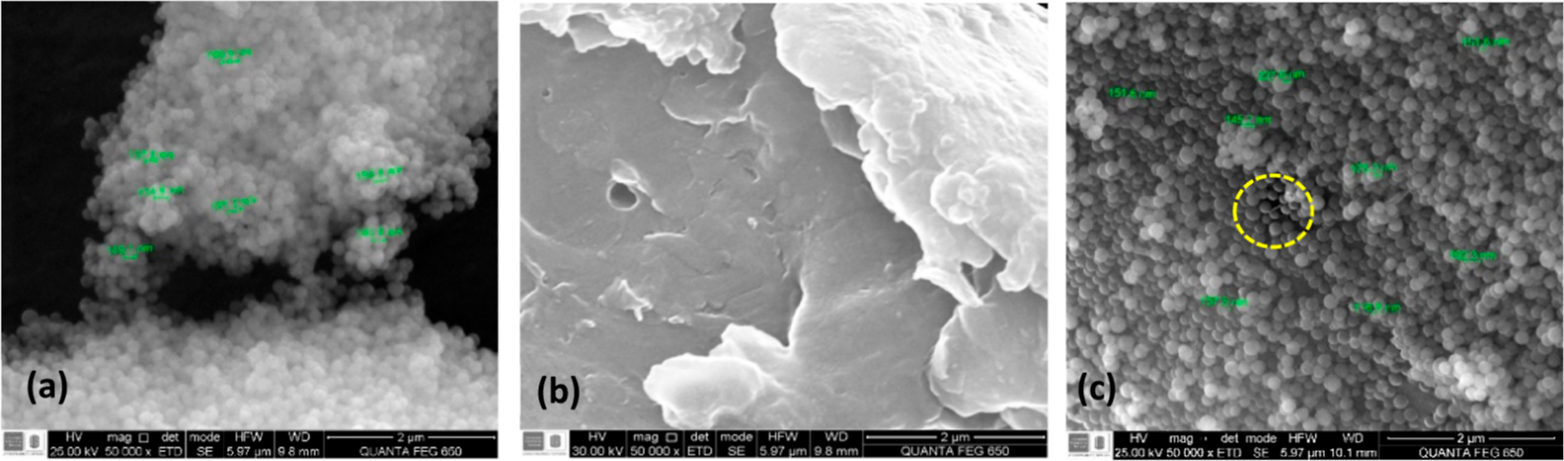
SEM micrographs of (a) nSiO_2_-APTES, (b) HPAM, and (c) CSNH-AC at 50,000X.

The SEM micrographs of CSNH-AC, HPAM, and nSiO_2_-APTES are presented in Figure 3. As shown in the images, the nSiO_2_–APTES have a spherical morphology and size of 150 nm (Figure 3a). The HPAM polymer has an amorphous morphology (Figure 3b), while the nanohybrid (Figure 3c) exhibits a well-formed structure with the NPs attached to the polymer at specific sites. The micrograph of the nSiO_2_ particles used to synthesize the nSiO_2_–APTES are not shown in Figure 3, but they have a spherical morphology and size of 85 nm.

**Figure 4:**
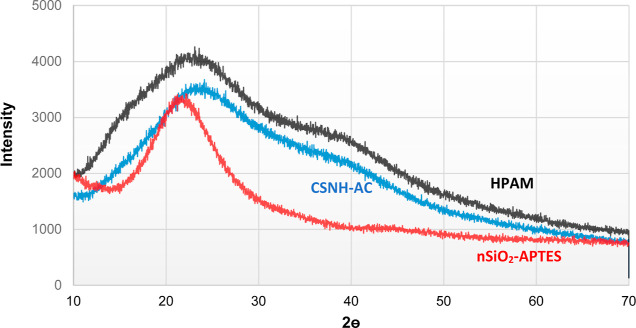
XRD patterns of HPAM, nSiO_2_-APTES, and CSNH-AC.

Figure 4 shows the diffractograms of nSiO_2_-APTES, CSNH-AC, and HPAM. The spectra of HPAM exhibit two broad halo peaks located at 2θ values of 23 and 40°. The spectra of the nSiO_2_-APTES exhibit a broad peak centered at around 2θ = 21.6°. Upon hybridization of the nSiO_2_-APTES with HPAM, this peak signal shifted to higher 2θ values. This was attributed to the attachment of the organic functional groups of the polymer onto the surface of NPs, which tends to reduce the scattering power of the amorphous silica.

In conclusion, all XRD patterns are typical of amorphous materials because the atoms are randomly distributed in three-dimensional space. In this case, the X-rays were scattered in many directions, giving rise to a halo distributed over a wide range of 2θ, not following Bragg’s Law.

#### 3.1.2 ATR–FTIR Results

**Figure 5:**
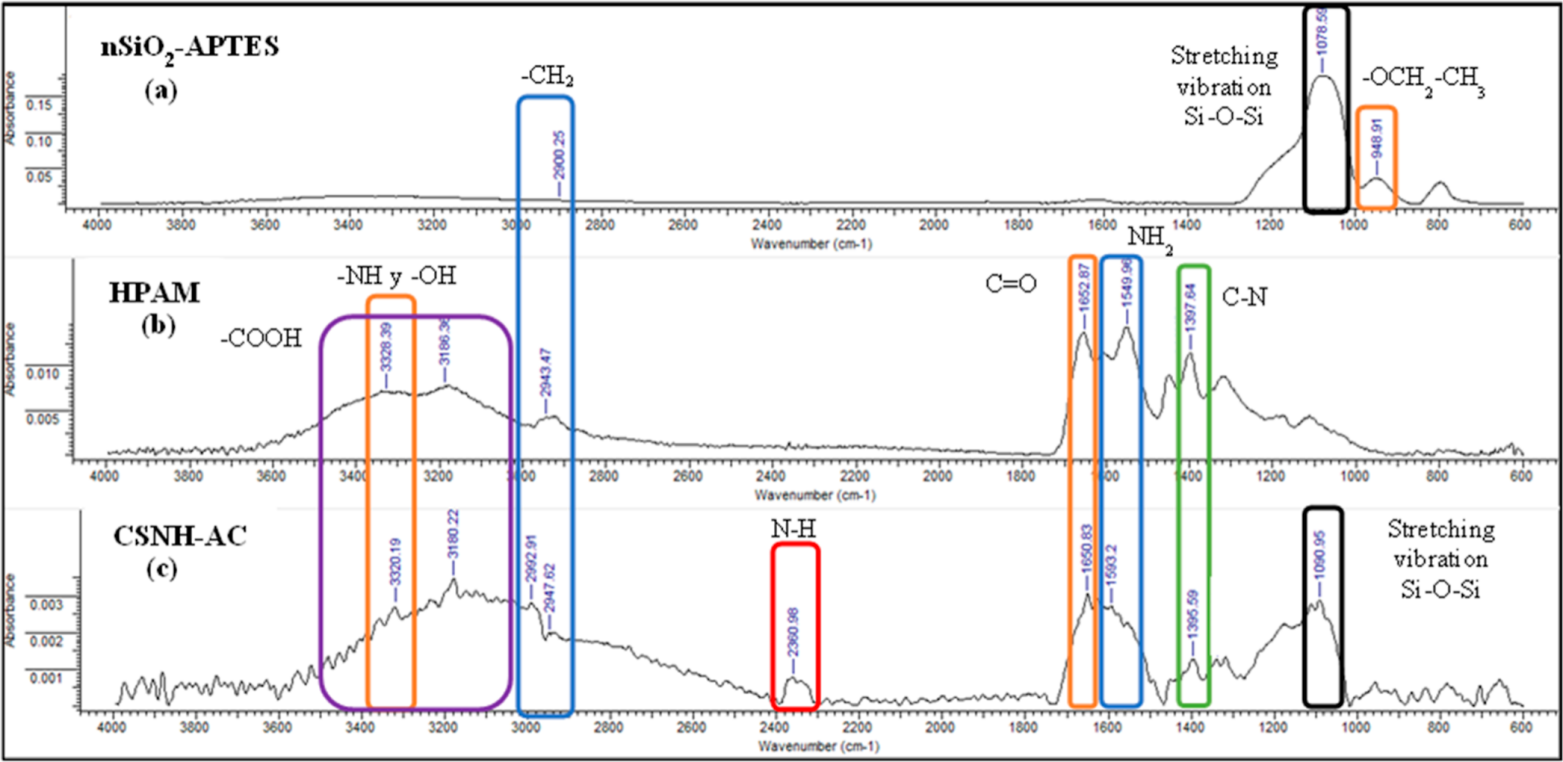
Infrared (IR) spectra of (a) nSiO_2_-APTES, (b) HPAM, and (c) CSNH-AC.

The FTIR spectra of nSiO_2_-APTES, HPAM, and CSNH-AC are compared in Figure 5. The characteristic peaks that confirm the nanohybrid formation are 3320 cm^–1^, which is related to the –NH stretching vibration and –OH stretching vibration.^68,69^ Other stretching vibrations identified are those attributed to –CH_2_ (2947 cm^–1^), C=O (1650 cm^–1^), C–N (1395 cm^–1^), and Si–O–Si (1090 cm^–1^).^70^ Furthermore, the presence of a secondary amide in the nanohybrid structure is confirmed by the peak at 2360 cm^–1^, which represents the covalent bond between the nSiO_2_-APTES and HPAM.

#### 3.1.3 TGA Results

**Figure 6:**
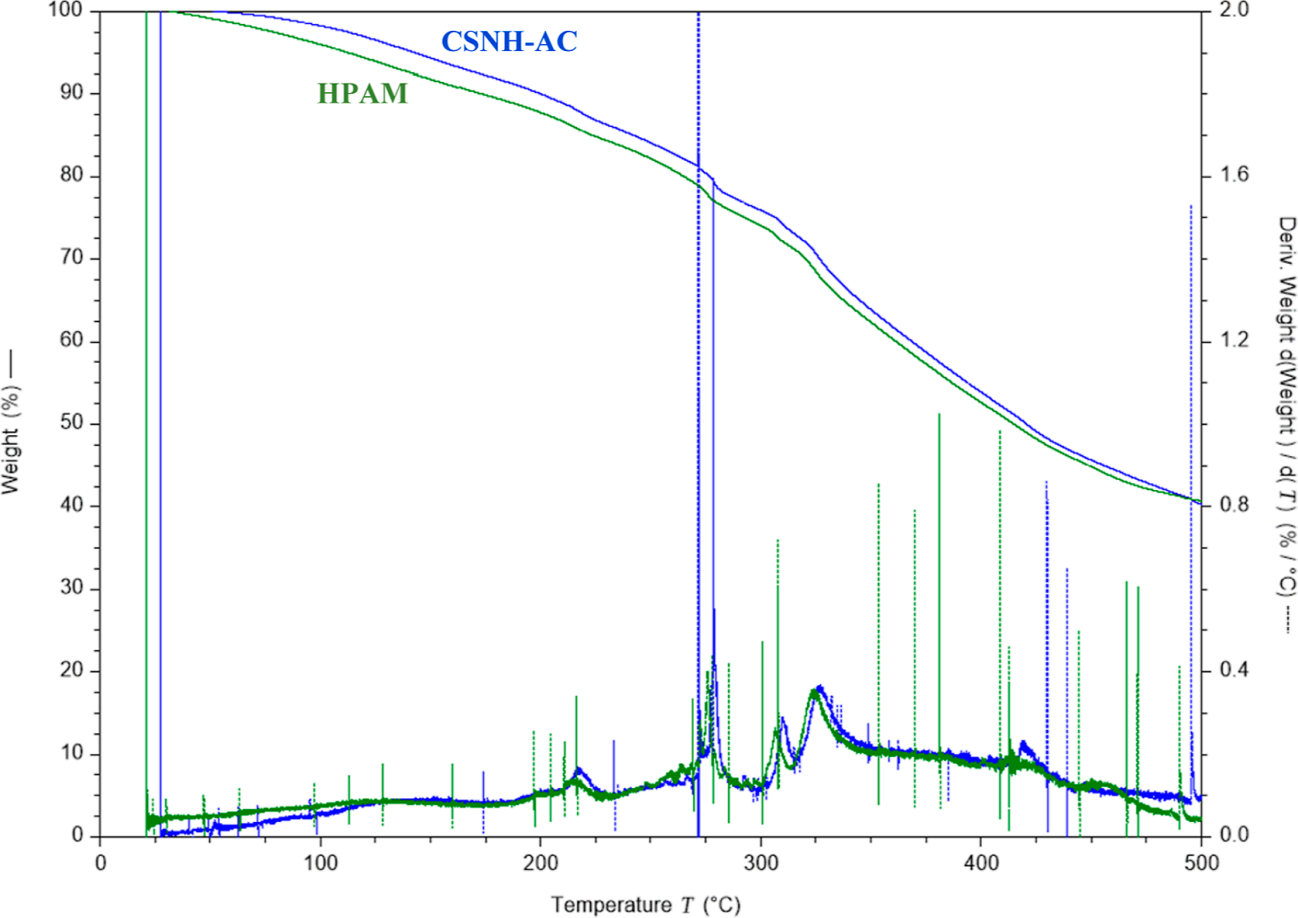
Thermograms of HPAM and CSNH-AC (heating rate of 10 °C/min under a nitrogen atmosphere).

TGA curves of CSNH-AC and HPAM are displayed in Figure 6. Both curves have three stages according to the peaks associated with the mass changes, which were identified as stage 1, from room temperature to 270 °C; stage 2, between 270 and 350 °C; and stage 3, >350 °C. The weight loss in stage 1 was 18% for HPAM and 21.1% for CSNH-AC, corresponding to the remaining adsorbed water or volatile solvents in each sample. The weight loss in stage 2 was 10.5% for HPAM and 9.9% for CSNH-AC, and it was assigned to the thermal decomposition of the amide and carboxylate groups of the polymer. Stage 3 corresponds to the decomposition of the C–C bonds from the HPAM backbone.^71^

In our previous work,^72^ it was reported that the weight loss between 350 and 600 °C of the nSiO_2_-APTES was 2.8%, which was attributed to the thermal decomposition of the aminopropyl groups. This weight loss is not significant in comparison to that reported for the HPAM and the CSNH-AC (>20%) in the same conditions.

### 3.2 Nanopolymer Sol and Polymer Solution Characterization

#### 3.2.1 Rheology

**Figure 7:**
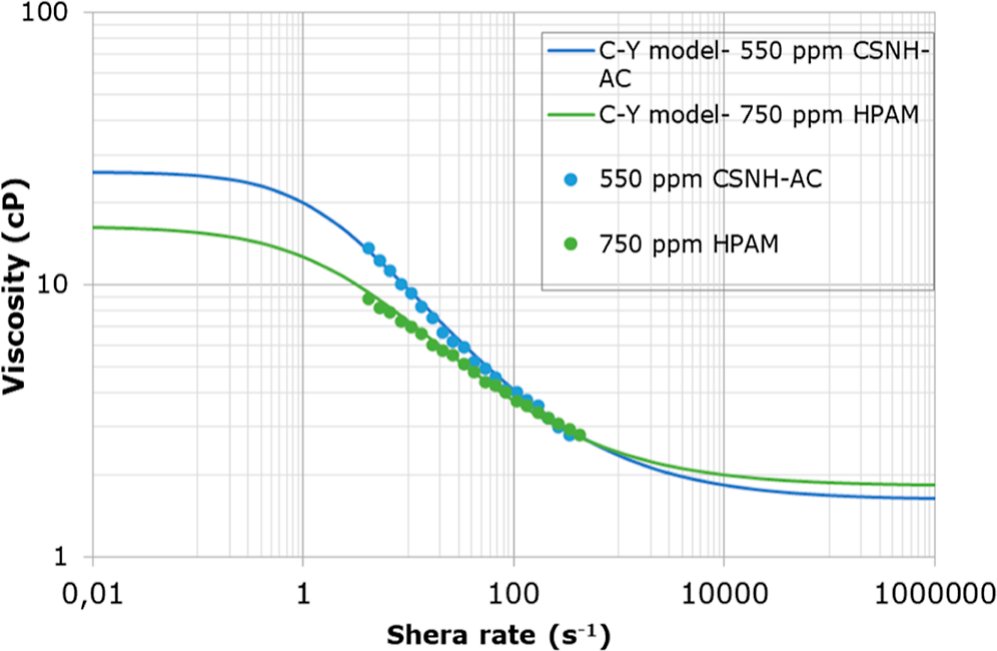
Log–log plots of 750 ppm of HPAM solution and 550 ppm of CSNH-AC nanohybrid viscosity at 56 °C.

As stated earlier, the viscosity data of the HPAM solution and the nanopolymer sol (Figure 7) follow the Carreau–Yasuda model. The model parameters are presented in Table 5. The nanohybrid sol exhibited slightly higher viscosities at shear rate values below 100 s^–1^ than the HPAM solution at a lower concentration due to the NP/polymer interaction.^73^ At higher shear rates, the viscosity of both solutions drops until they reach brine viscosity (infinite viscosity of the Carreau–Yasuda model).

**Table 5:** Viscosity Carreau–Yasuda Parameters for the HPAM Solution and the CSNH-AC Nanohybrid at 56 °C

parameter/sample	550 ppm of CSNH-AC	750 ppm of HPAM
η_o_ [mPa·s]	26.052	16.445
η_∞_ [mPa·s]	1.622	1.819
λ	0.723	0.458
α	1.018	1.598
*n*	0.468	0.465

### 3.3 Dynamic Adsorption and IPV

**Figure 8:**
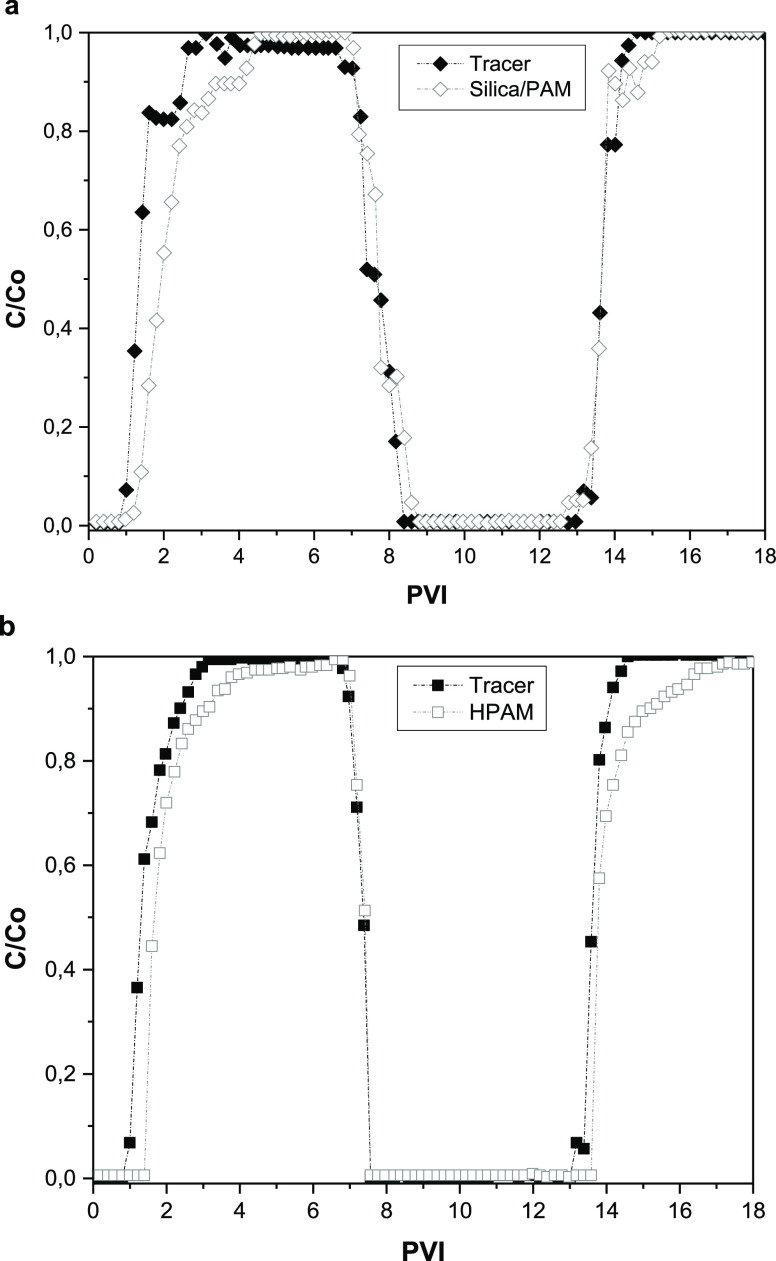
Breakthrough curves of (a) 550 ppm nanohybrid, (b) 750 ppm of HPAM and tracer slugs (30 ppm KSCN) at Sw = 1 and 1 ft/day.

The breakthrough curves of the HPAM, the nanopolymer sol, and the tracer (KSCN) slugs are shown in Figure 8. The first nanopolymer sol slug had a later breakthrough than the tracer (Figure 8a), showing that nanohybrid retention predominates over the effect of the IPV. The retention occurs by mechanical entrapment, which is the primary mechanism for the slow recovery of the nanohybrid after the breakthrough.^74^ Also, the breakthrough of the first and second slugs of the HPAM solution happened later than the tracer due to polymer retention (Figure 8b). For the HPAM solution and the nanohybrid sol, the second tracer slugs breakthrough earlier than the first ones because the polymer retention in the first injection reduced the available pore volume for the tracer.

The breakthrough time difference method was used to measure the IPV of the HPAM solution and the nanopolymer sol (eq 3). This method provides better accuracy in determining IPV than the areal difference when mechanical entrapment occurs during the core flooding test.^74^ The nanopolymer sol exhibits higher mechanical entrapment and IPV than the HPAM solution (Table 6) due to its tridimensional network conformation. Also, these parameters could be affected by the low permeability of the rock used in the experimental test.^75^

**Table 6:** Adsorption and IPV of the Nanohybrid and HPAM at Sw = 1

sample	concentration (mg/L)	μ@7.3 s^–1^	adsorption (μg/g)	IPV (%)
HPAM	750	8	9.2	10
nanohybrid	550	10.9	29.7	14.5

### 3.4 Mobility (RF) and Permeability (RRF) Reduction

**Figure 9:**
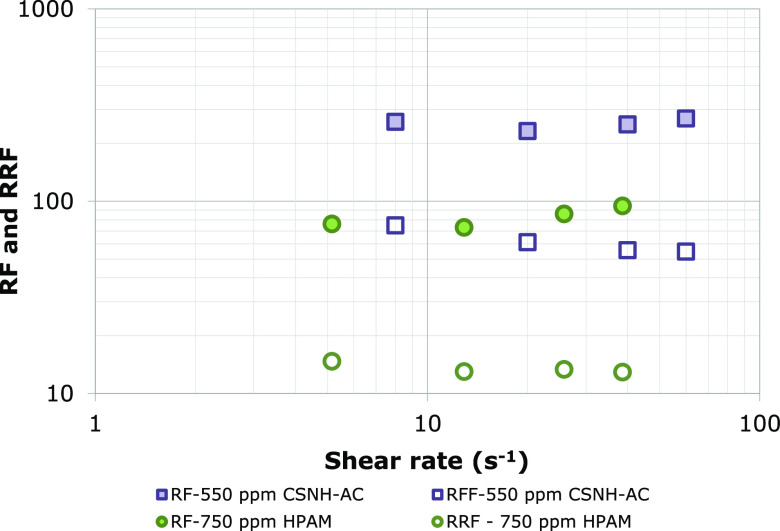
RF and RRF of 750 ppm of HPAM solution and 550 ppm of nanopolymer sol injection.

**Figure 10:**
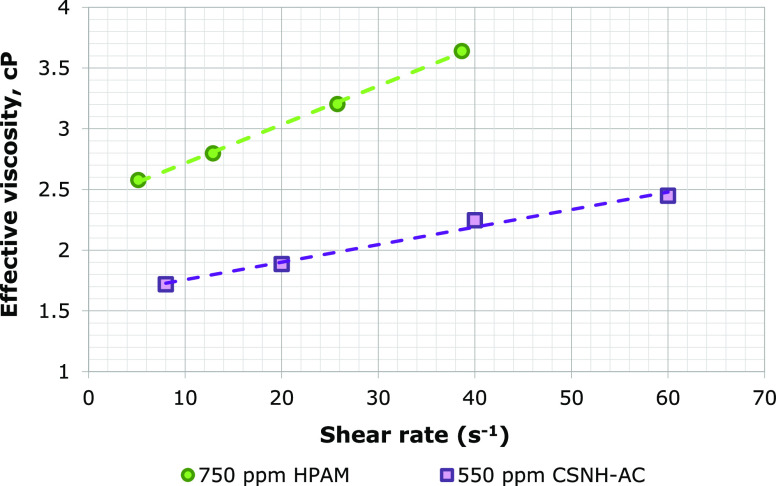
Effective viscosity of the 750 ppm of HPAM solution and 550 ppm nanopolymer sol.

The RF and RRF values of the HPAM solution and the nanopolymer sol at different shear rates were calculated from eqs 1 and 2 and are shown in Figure 9a,b. The curve of effective viscosity was obtained by eq 7 and is presented in Figure 10. For the HPAM solution, the RF gradually increases with the increase in shear rate, while the RRF slightly decreases and tends to balance. It has been previously reported that the increase in the injection rate of the polymer solution (shear rate) produces the elastic deformation of the polymer molecules by hydrodynamic forces,^32^ leading to an increase in the effective viscosity and the RF.^66^ In contrast, the increase in the injection rate of the chase water causes a reduction in the RRF due to the scouring of the retained polymer molecules in the porous media. The nanopolymer sol’s RF and RRF values are greater than those of the HPAM solution but exhibit the same trend with an increase in the injection rate. This can be attributed to the strong flow resistance of the nanohybrid (high retention in the porous media).

Due to the high RRF values obtained for both products, some changes in the methodology should be considered, such as the use of higher permeability rocks, the injection of the polymer/nanohybrid until *C*/*C*_o_ = 0,5 to prevent filter cake formation, and an increase in the pore volumes of brine injected in the postflush. It could improve the estimation of these parameters, which are vital to the proper design of field-scale polymer projects.

### 3.5 History Matching

**Figure 11:**
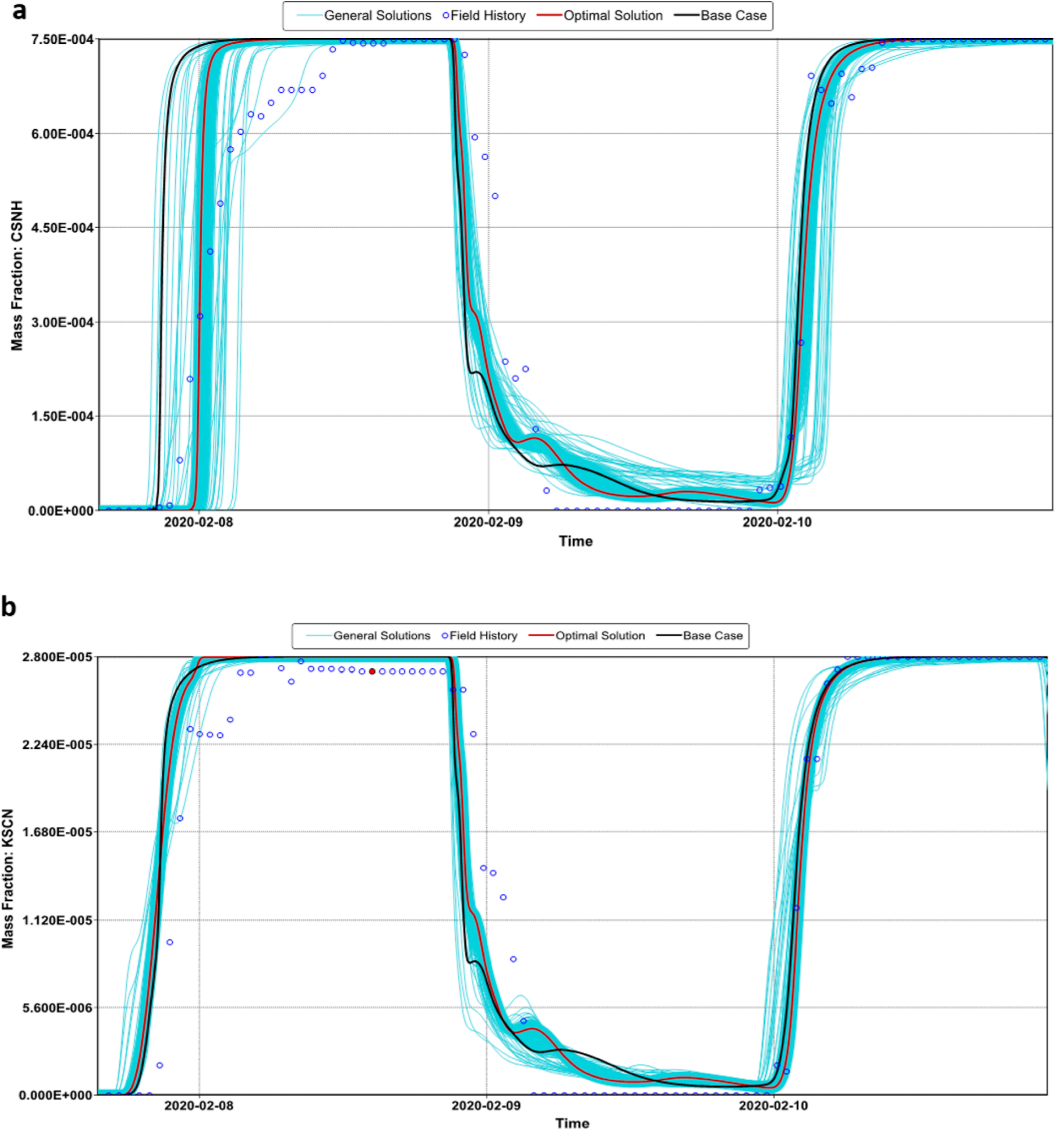
Experimental and predicted breakthrough curves of (a) 550 ppm of nanopolymer sol and (b) 30 ppm of KSCN at Sw = 1.

**Figure 12:**
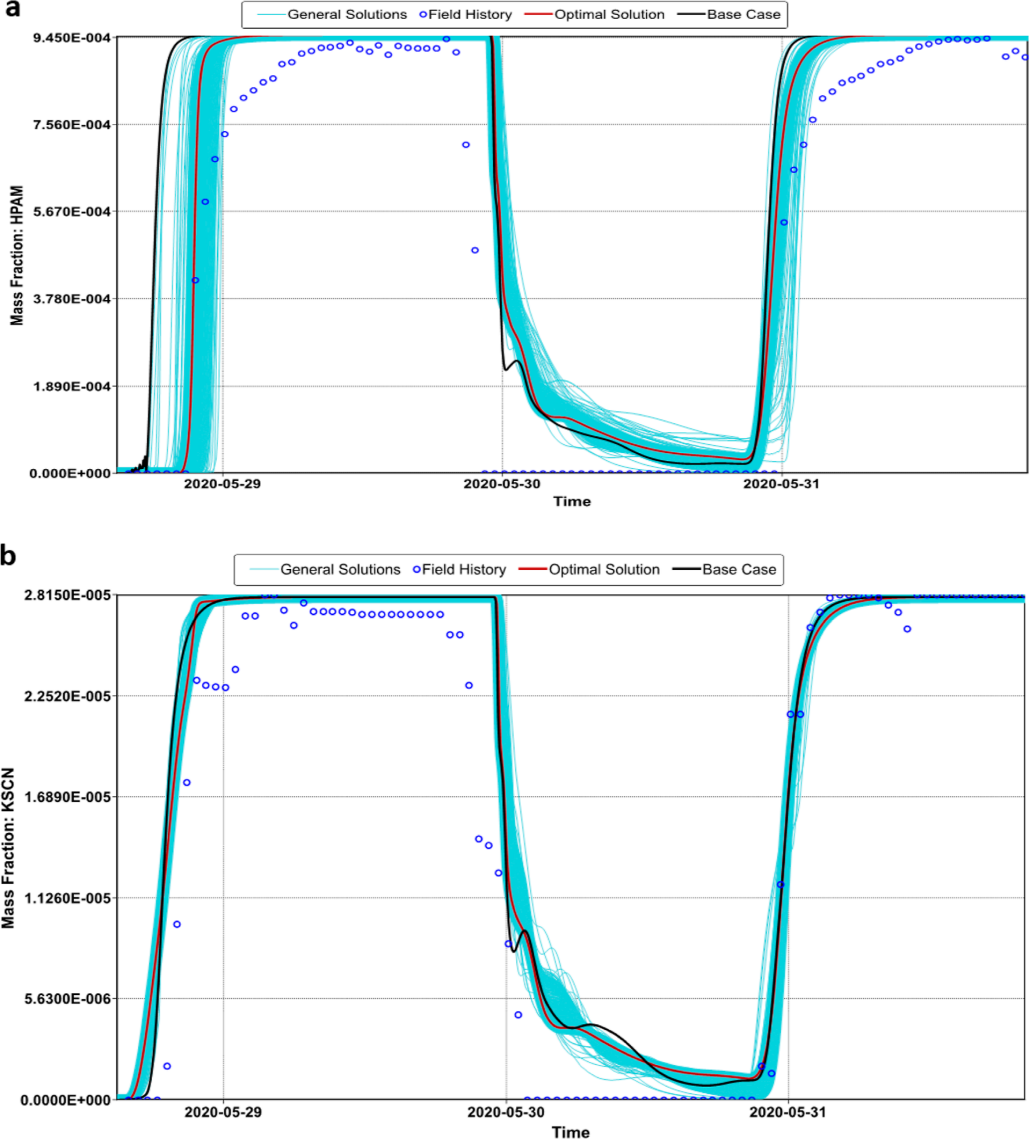
Experimental and predicted breakthrough curves of (a) 750 ppm of HPAM solution and (b) 30 ppm of KSCN at Sw = 1.

**Figure 13:**
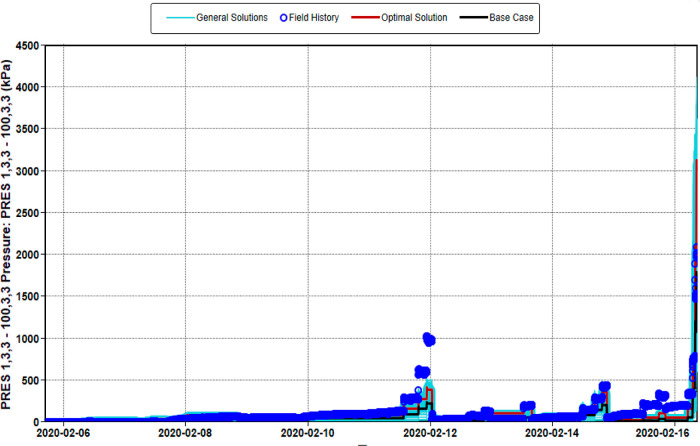
Experimental and predicted pressure drop curves for the injection of 550 ppm of nanopolymer sol and 30 ppm KSCN.

The objective functions for the history matching were the pressure drops recorded during the core flooding tests and the breakthrough curves shown in Figure 8. Figures 11 and 12 show the modeling of the laboratory production curves of the nanopolymer sol and the HPAM solution, respectively. 500 possible solutions were run by the probabilistic simulator (CMG-CMOST). Figure 13 shows the history matching of the pressure drop during the CSNH-AC flooding at the laboratory scale. Table 7 presents the parameters used in the probabilistic simulation for history matching. It was observed that the best solution (red line) accurately predicts the breakthrough of the first slug of the nanopolymer sol and the HPAM. However, all solutions predicted an anticipated tracer breakthrough. Two reasons can be attributed to this result: (1) the use of a homogeneous conceptual model to represent the average properties of the core sample (porosity and permeability) presents limitations for reproducing the possible heterogeneities in the core plugs, and (2) the tracer concentration was not determined in real-time, causing a difference between the actual breakthrough time and the reported one.

**Table 7:** Parameters Used in the Probabilistic Simulation for History Matching

parameter		unit	range
maximum adsorption	ADMAXT	mg/g roca	0.01–0.5
residual adsorption	ADRT	%	50–100
water relative permeability at Sor	KRWIRO	fr	0.4–1
RRF	RRF		1–12
accessible pore volume	VPA = (1 – VPI)	fr	0.7–1
horizontal permeability multiplier	PERMI		0.4–1.2
vertical permeability multiplier	PERMK		0.1–1.0

The best-fit parameters for the three target functions of both core flooding tests are presented in Table 8. The calculated maximum adsorptions for the CSNH-AC and HPAM are significantly higher than those obtained in the laboratory tests. Polymer adsorption is considered a reversible process that depends on the polymer concentration, rock composition, salinity, and hardness. In the numerical simulation, the reversibility of the adsorption is represented by two modeling parameters: maximum and residual adsorption. The maximum adsorption includes mechanical entrapment, hydrodynamic retention, and chemical adsorption.^2,67^ When flow conditions change in porous media (i.e., velocity, flow direction, and polymer concentration), some retained chemicals are released^29^ but another amount remains adsorbed (residual adsorption) by chemical and/or physical interactions between the polymer backbone and the rock surface.^68^ For this reason, the estimated and measured adsorption values differ.

**Table 8:** Model Parameters for Polymer and Nanohybrid Flooding

parameters	CSNH-AC	HPAM
maximum adsorption, μg/g_rock_	158	125
residual adsorption, μg/g_rock_	17	98
IPV, %	10.4	7.78
RRF	7.37	1.25

Lower values of IPV and RRF than those obtained by the laboratory test were predicted by the history matching of CSNH-AC and HPAM. However, the calculated residual adsorption that fits the HPAM model is higher than the laboratory value. RRF, IPV, and desorption values depend on the core heterogeneity. Adsorption can reduce the flow path, leading to a reduction in effective permeability.^32,69^ Therefore, if adsorption decreases, IPV and RRF decrease. Since the history-matching data reproduced the performance of the chemical slugs in the laboratory tests, they were used to forecast the oil production in the sector model (Figure 2).

**Figure 14:**
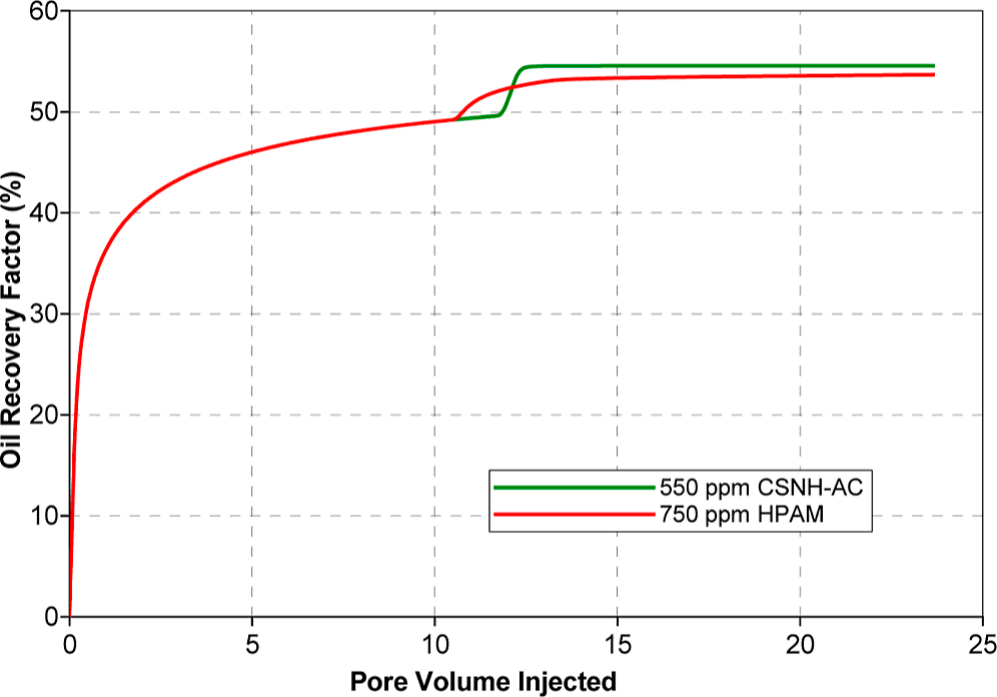
Comparative oil recovery factor of the nanopolymer sol and HPAM flooding.

The model parameters presented in Table 8 were used to predict the displacement efficiency of the nanopolymer sol and the HPAM solution (Figure 14) in the laboratory-scale simulation model (model 1, Table 3). The waterflooding was performed by injecting 10 PV. Then, 0.3 PV of polymer solution (750 ppm) or nanopolymer sol (550 ppm) was injected, followed by 23 PV of water. The incremental recovery factors (compared to the waterflooding) of the WF/HPAM/WC and the WF/CSNH-AC/WC schemes were 4.1 and 5.4%, respectively. An acceleration of the oil production was observed for the HPAM injection, although the final recovery factor of the nanopolymer sol was 1.3% higher at a lower concentration (Figure 14, green line). This oil recovery is comparable to that obtained in the laboratory tests previously reported by Corredor et al. (2021),^73^ where the displacement experiments showed that the nanopolymer sol increased the cumulative oil recovery by 2.2% OOIP compared to the HPAM solution. These results were attributed to the reduction of the capillary forces, the increment of the viscous forces,^73^ and the contact of unswept oil areas due to the piston-like displacement of the chase water after the nanopolymer sol injection.

The differential pressures obtained by numerical simulation and the laboratory displacement tests^73^ were similar. The differential pressures estimated by numerical simulation during the CSNH-AC and HPAM injections were 34.8 and 9.1 psi, respectively. Meanwhile, the maximum differential pressures reached on laboratory tests were 21.8 and 10.2 psi when 0.4 PV of CSNH-AC (550 ppm) and HPAM (750 ppm) were injected into the porous media, respectively. The results of the CSNH-AC injection suggest that the nanohybrid was able to reduce the water permeability (log jamming),^70^ allowing the nanopolymer sol to contact unswept zones and displace the oil trapped in the porous media. However, special attention should be paid to the injectivity of the nanopolymer sol.

**Figure 15:**
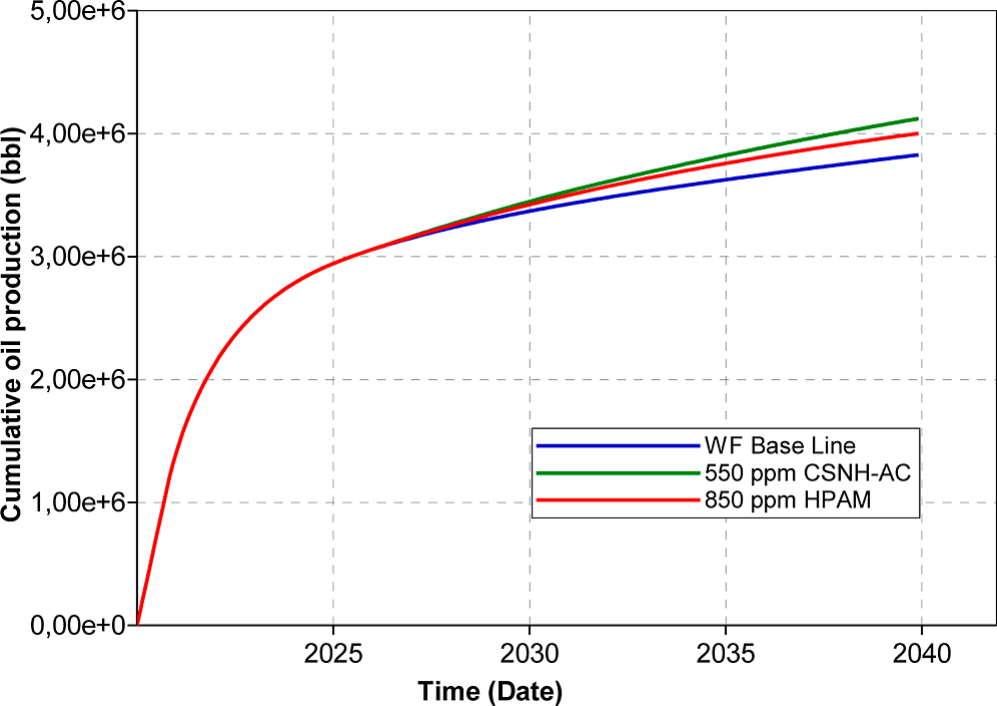
Comparative cumulative oil production of water and HPAM/nanohybrid flooding.

The model presented in Figure 2 and Table 4 was used to perform a field-scale simulation. The HPAM concentration was increased from 750 to 850 ppm to reach the target apparent viscosity of 5 cP in porous media, while the CSNH-AC concentration was kept at 550 ppm. Figure 15 shows the predicted cumulative oil production for water and the HPAM/nanohybrid injection. The injection of the nanopolymer sol and the HPAM solution (0.1 PV) increased the oil production by 295,505 and 174,465 barrels, respectively, compared to waterflooding. This will represent an incremental recovery factor of 11.3% for the nanopolymer sol and 6.7% for the HPAM solution. Increasing the concentration of the CSNH-AC from 550 to 1200 ppm will produce an additional 60,550 barrels of oil (2.3% of incremental recovery factor). Instead, the incremental oil production from increasing the HPAM concentration from 850 to 1500 ppm will be 35,500 barrels (1.35% of incremental recovery factor). Even after injecting 1500 ppm of HPAM, the incremental oil production is lower than that of 550 ppm of CSNH-AC. Nonetheless, the optimal chemical concentration for a field application should be established based on the operational conditions (i.e., injectivity) and the economic feasibility.

## 4 Conclusions

This study reports the results of the retention experiment and numerical simulation of the displacement efficiency of a SiO_2_/HPAM nanohybrid. The nanohybrid was characterized by attenuated total reflection–Fourier transform infrared spectroscopy (ATR–FT-IR), FEG–SEM, XRD, and TGA. The results showed that the nanohybrid exhibits better rheological behavior than the HPAM solution at a lower concentration. The RF and RRF values of both samples are shear-dependent. The RRF values decrease by increasing the shear rate (injection rate) due to the scouring of the retained polymer molecules in the porous media by the chase water. In contrast, the RF values increase with the increase in shear rate due to the deformation of the adsorbed polymer/nanohybrid layer by hydrodynamic forces. The nanohybrid exhibited greater retention and IPV than the HPAM solution due to its tridimensional network conformation and because it was injected in a lower permeability core. The incremental recovery factors predicted by the field-scale simulation were 11.3 and 6.7% for the nanopolymer sol and the HPAM solution (as compared to waterflooding), respectively. More oil production with less chemical injection may widen the applications of nanohybrids for the EOR process, but further experiments should be performed.

## Nomenclature

APTES: 3-aminopropyltriethoxysilane
*C*: polymer concentration on the effluents
*C*_o_: initial polymer concentration
CSNH-AC: SiO_2_–PAM nanohybrid
EOR: enhanced oil recovery
FEG–SEM: field emission gun–scanning electron microscopy
FTIR: Fourier-transform infrared spectroscopy
HPAM: hydrolyzed polyacrylamide
IPV: inaccessible pore volume
K: absolute permeability
KCl: potassium chloride
NaCl: sodium chloride
NP: nanoparticle
*Q*: flow rate
PV: pore volume
PVI: pore volume injected
*R*_p_: porous radius
RF: resistance factor
RRF: residual resistance factor
TGA: thermogravimetric analysis
XRD: X-ray diffraction
Δ*P*_w_: pressure drop during brine injection
Δ*P*_wp_: pressure drop during brine injection after polymer flooding
Δ*P*_p_: pressure drop during polymer or nanopolymer sol injection.

## Greek Letters

γ̇: shear rate
Φ: porosity
α: formation shape factor
μ: viscosity
